# Supplementary material for: Ecological divergence of wild birds drives avian influenza spillover and global spread
Source: PLoS Pathog. 2022 May 19;18(5):e1010062. doi: 10.1371/journal.ppat.1010062 (PMC9119557; doi:10.1371/journal.ppat.1010062)
Supplement: S1 Text — The left panels indicate the branching pattern and estimated divergence times of lineages (blue bars: 95% highest posterior density). The right panels indicate geographic origin of viruses (color-coded branches) estimated using a phylogeographic discrete trait model with Bayesian inference (Beast v10.4). (DOCX) [file ppat.1010062.s001.docx]

**S1 Text. Phylodynamics of H13 (A), H16 (B) and highly pathogenic H5 (C) subtypes based on the hemagglutinin gene time-calibrated phylogeny.** The left panels indicate the branching pattern and estimated divergence times of lineages (blue bars: 95% highest posterior density). The right panels indicate geographic origin of viruses (color-coded branches) estimated using a phylogeographic discrete trait model with Bayesian inference (Beast v10.4).


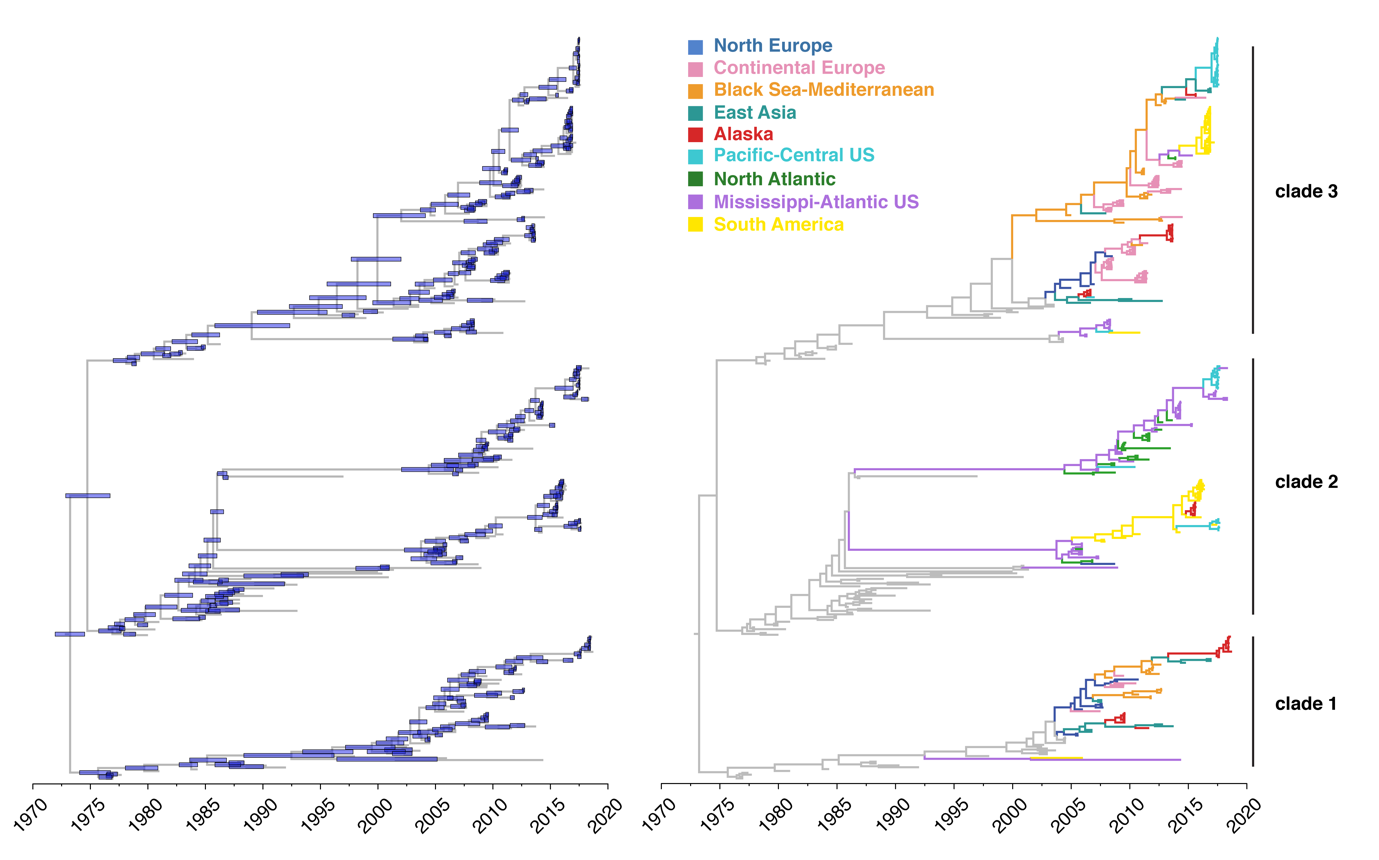


­­

A) H13


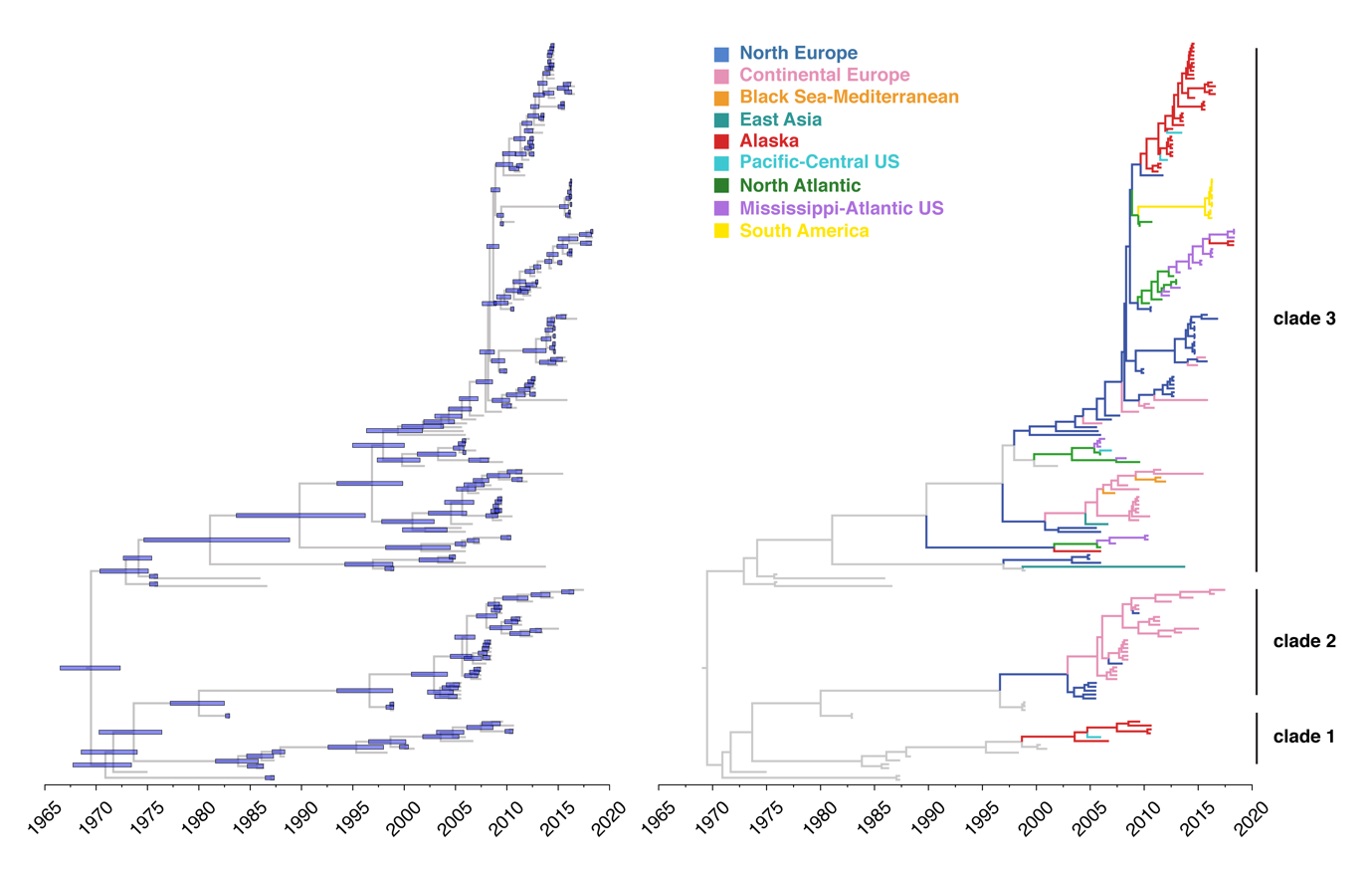
­­­­­­

B) H16


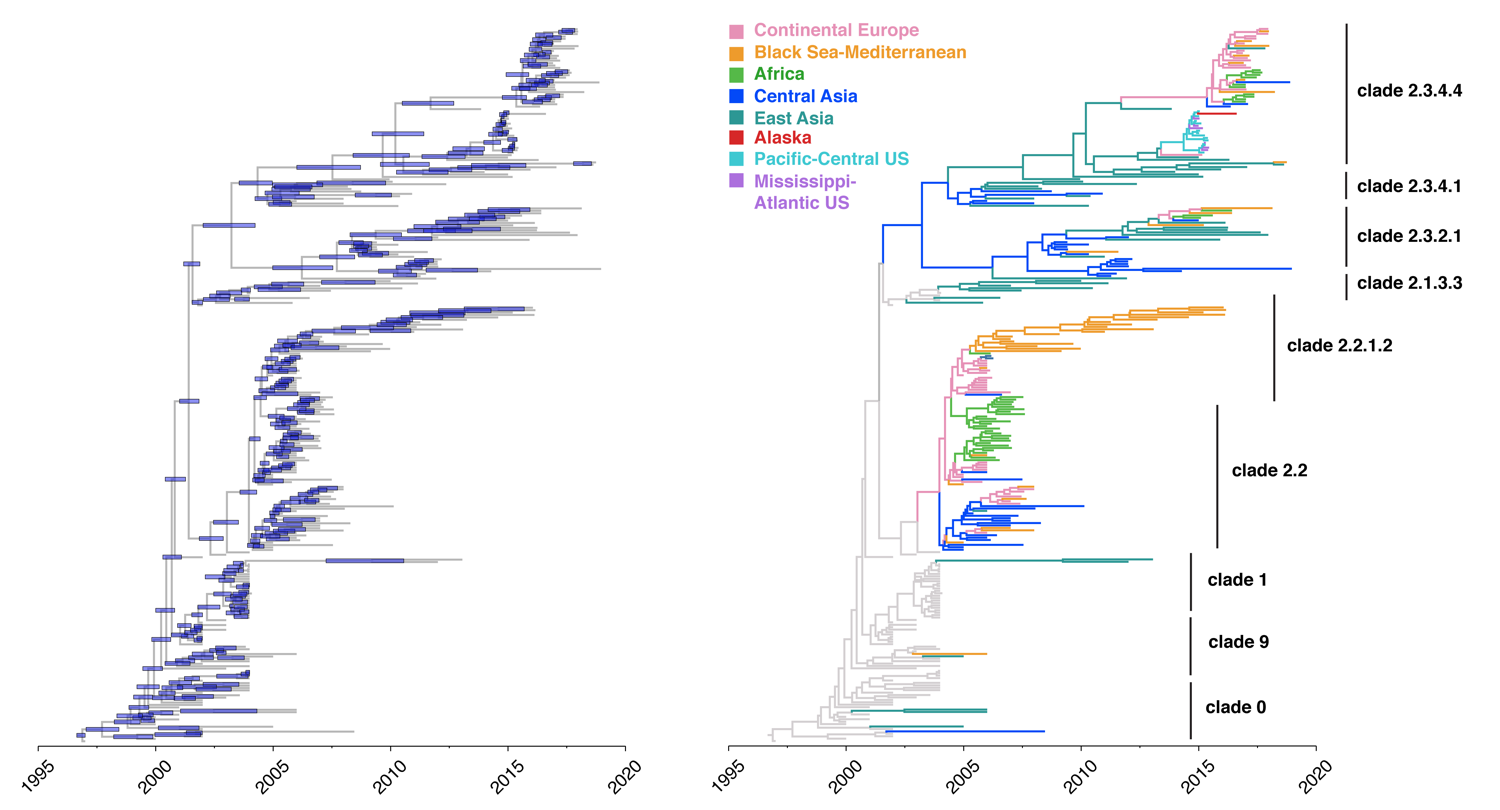


C) HPAI H5
