# Supplementary material for: Ecological divergence of wild birds drives avian influenza spillover and global spread
Source: PLoS Pathog. 2022 May 19;18(5):e1010062. doi: 10.1371/journal.ppat.1010062 (PMC9119557; doi:10.1371/journal.ppat.1010062)
Supplement: S2 Text — A & B) H16 emerged from two geographically-distinct clades (blue and purple) that predominate, with introductions of H13 occurring periodically from East Asia (green) and South America (yellow). Geographic origins were determined by most recent common ancestor (MRCA) phylodynamic analysis. B) The temporal distribution of H13 and H16 virus lineages mapped onto two-dimensional space. The temporal distribution of the strains and subtypes in Cordova, Alaska indicates that the two clades of H16 from Northern Europe and the Mississippi-Atlantic US rarely co-circulate, suggestive of geographic structuring and high antigenic cross-reactivity. In contrast, the temporal overlap between H16 (Mississippi-Atlantic clade) and H13 (East Asian clades) is indicative of low competition or antigenic cross-reactivity. (DOCX) [file ppat.1010062.s002.docx]

**S2 Text.** **Temporal pattern of H13 and H16 circulation in gulls over a 10-year period in Cordova, Alaska**. A & B) H16 emerged from two geographically-distinct clades (blue and purple) that predominate, with introductions of H13 occurring periodically from East Asia (green) and South America (yellow). Geographic origins were determined by most recent common ancestor (MRCA) phylodynamic analysis. B) The temporal distribution of H13 and H16 virus lineages mapped onto two-dimensional space. The temporal distribution of the strains and subtypes in Cordova, Alaska indicates that the two clades of H16 from Northern Europe and the Mississippi-Atlantic US rarely co-circulate, suggestive of geographic structuring and high antigenic cross-reactivity. In contrast, the temporal overlap between H16 (Mississippi-Atlantic clade) and H13 (East Asian clades) is suggestive of low competition or antigenic cross-reactivity.


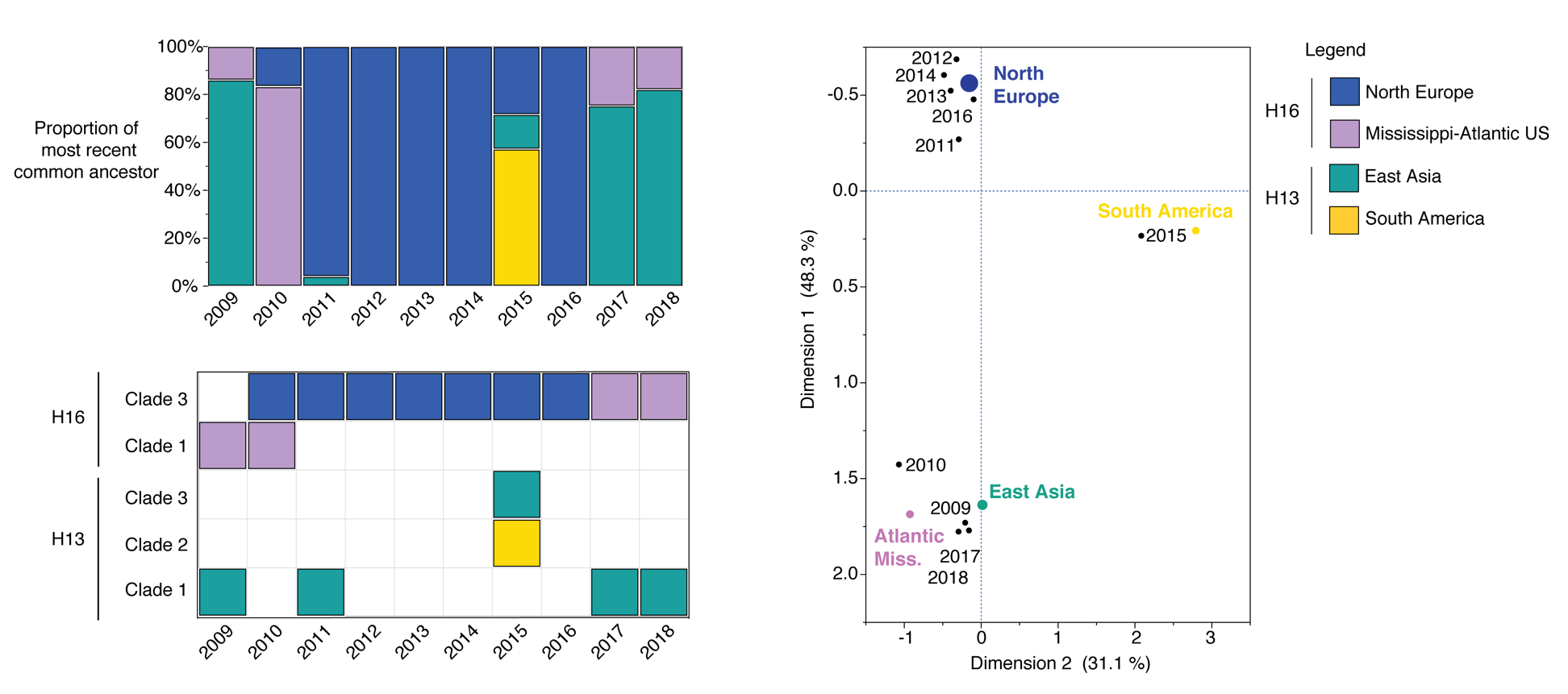


A)

C)

B)
