# Supplementary material for: Ecological divergence of wild birds drives avian influenza spillover and global spread
Source: PLoS Pathog. 2022 May 19;18(5):e1010062. doi: 10.1371/journal.ppat.1010062 (PMC9119557; doi:10.1371/journal.ppat.1010062)

**S3 Text. Transitions of the PB2 segment between HA subtypes circulating within the Cordova gull population in Alaska (2009-2018).** These estimates quantified reassortment and were based on a discrete trait model. As indicated by high Bayes factor support, H16 was a major source of the internal segment to H13 and to a lesser degree H13 acted as a source of PB2 to H16.


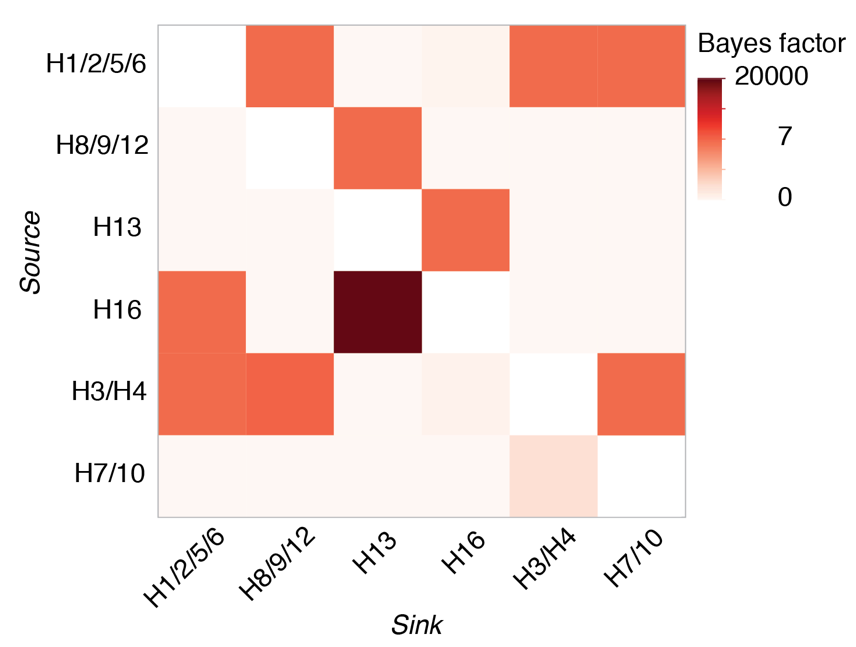

Supplement: S3 Text — These estimates quantified reassortment and were based on a discrete trait model. As indicated by high Bayes factor support, H16 was a major source of the internal segment to H13 and to a lesser degree H13 acted as a source of PB2 to H16. (DOCX) [file ppat.1010062.s003.docx]
