# Supplementary material for: Ecological divergence of wild birds drives avian influenza spillover and global spread
Source: PLoS Pathog. 2022 May 19;18(5):e1010062. doi: 10.1371/journal.ppat.1010062 (PMC9119557; doi:10.1371/journal.ppat.1010062)

**S4 Text. Spatial clustering method for influenza A virus sequences used to define ‘geoclusters’.** For each viral sequence of H13, H16, and H5 (low and highly pathogenic) we extracted centroid coordinates for the lowest administrative unit using Geonames.org. All sampling coordinates were input into a k-means cluster analysis to determine groupings of sampling sites based on geographic proximity rather than administrative or political boundaries. A total of 11 unique geoclusters were identified across subtypes that corresponded to geographic ‘traits’ used for subsequent phylodynamic analysis. The base map is made with Natural Earth (naturalearthdata.com).


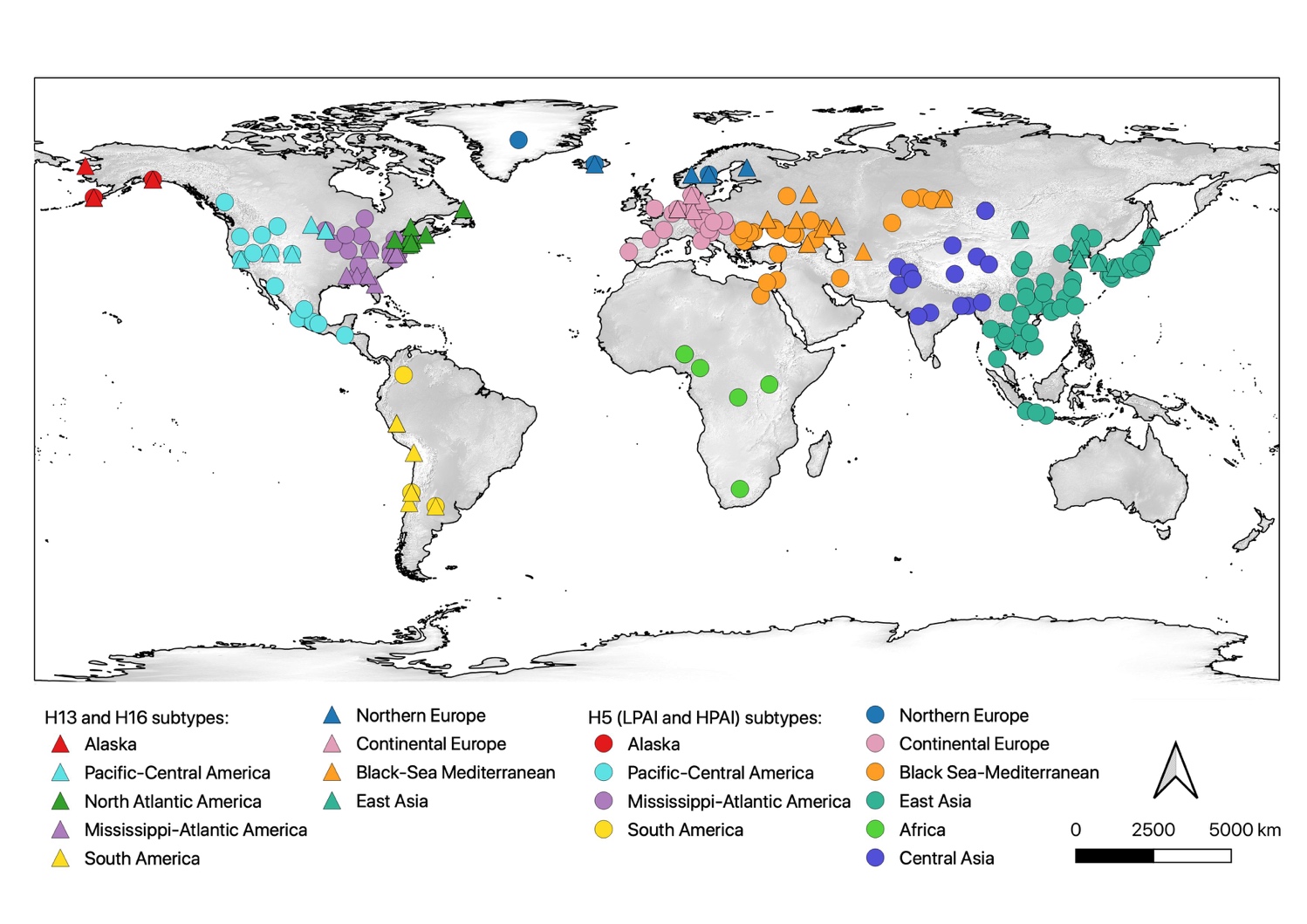

Supplement: S4 Text — For each viral sequence of H13, H16, and H5 (low and highly pathogenic) we extracted centroid coordinates for the lowest administrative unit using Geonames.org. All sampling coordinates were input into a k-means cluster analysis to determine groupings of sampling sites based on geographic proximity rather than administrative or political boundaries (implemented using JMP Pro, SAS). A total of 11 unique geoclusters were identified across subtypes that corresponded to geographic ‘traits’ used for subsequent phylodynamic analysis. The base map is made with Natural Earth (naturalearthdata.com). (DOCX) [file ppat.1010062.s004.docx]
