# Supplementary material for: Ecological divergence of wild birds drives avian influenza spillover and global spread
Source: PLoS Pathog. 2022 May 19;18(5):e1010062. doi: 10.1371/journal.ppat.1010062 (PMC9119557; doi:10.1371/journal.ppat.1010062)

**S5 Text. Downsampling method for influenza A virus sequences.** Datasets were stratified by location attributes (geocluster) and downsampled to ensure an equivalent number of samples for each the H13, H16 and HPAI H5 subtypes. For each geocluster (ie. Africa or East Asia), maximum likelihood phylogenetic trees were constructed using RAxML v8.2.12 (Stamatakis 2006) employing the GTRGAMMA substitution model. Each tree was downsampled to approximately 40 taxa while preserving the maximum amount of genetic diversity using the Phylogenetic Diversity Analyzer tool ([www.cibiv.at/software/pda](http://www.cibiv.at/software/pda)) following the approach of Trovao *et al.* (2015). This down-sampling approach was repeated for each subtype stratified by host traits.


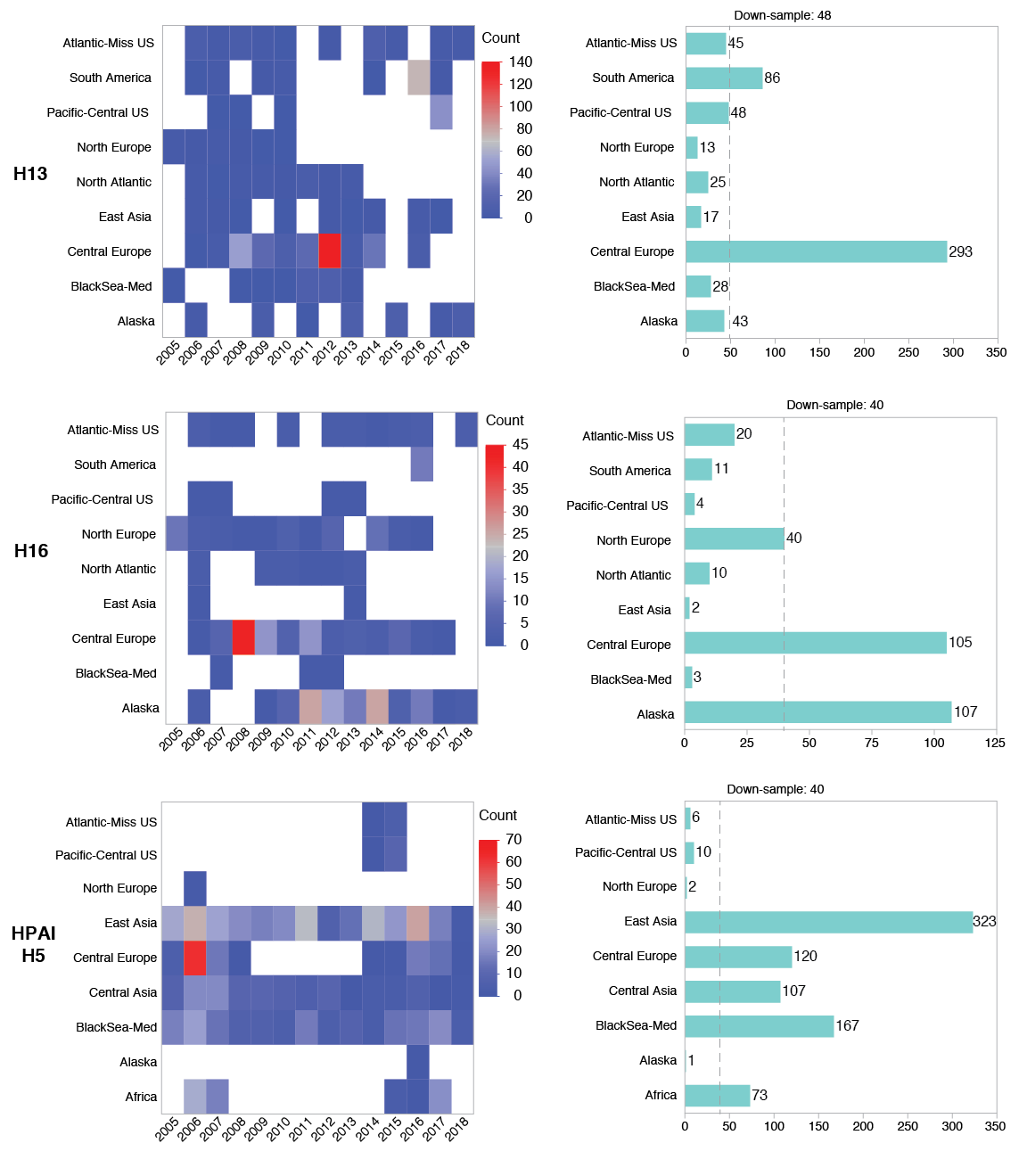


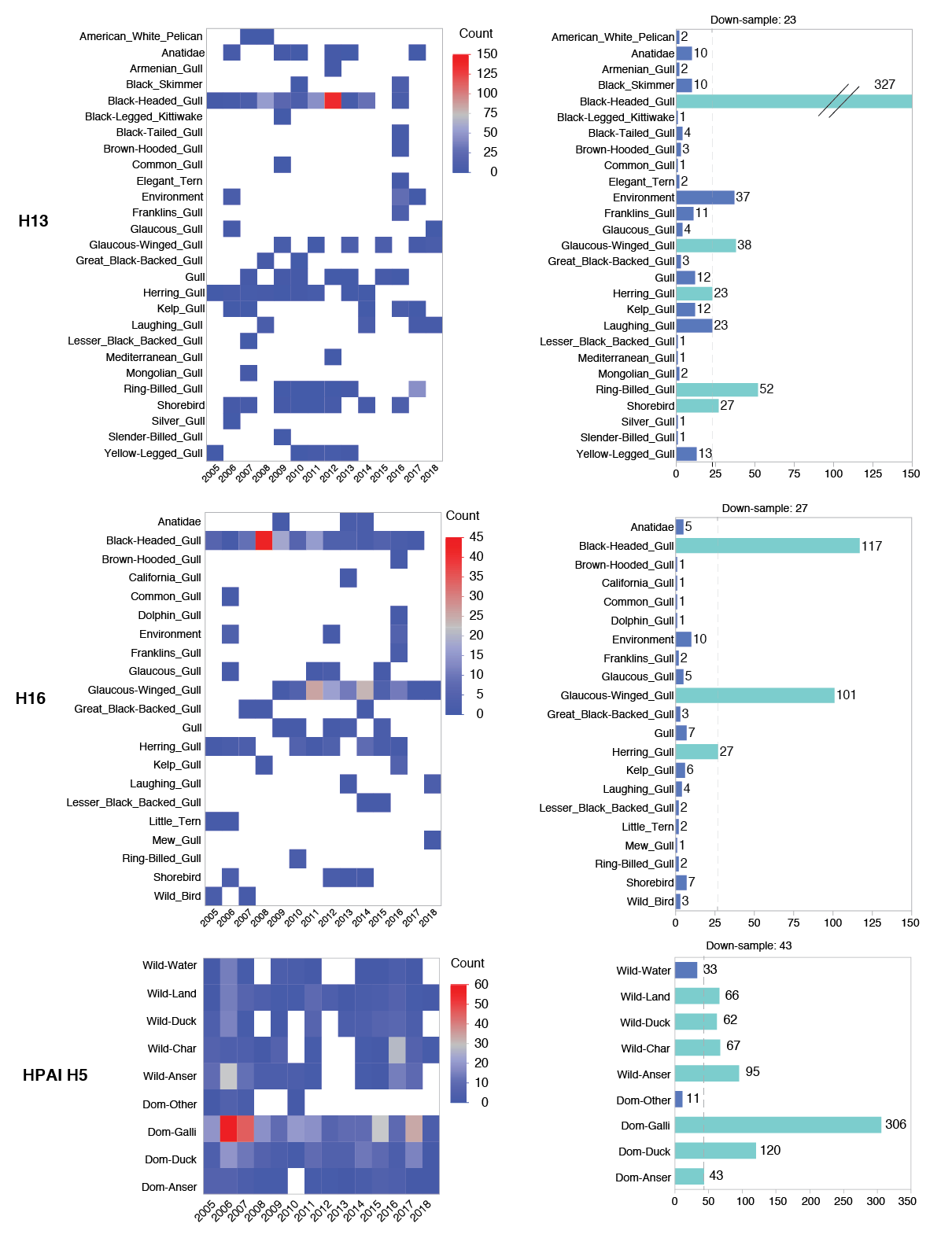

Supplement: S5 Text — Datasets were stratified by location attributes (geocluster) and downsampled to ensure an equivalent number of samples for each the H13, H16 and HPAI H5 subtypes. For each geocluster (ie. Africa or East Asia), maximum likelihood phylogenetic trees were constructed using RAxML v8.2.12 (Stamatakis 2006) using the GTRGAMMA substitution model. Each tree was downsampled to approximately 40 taxa while preserving the maximum amount of genetic diversity using the Phylogenetic Diversity Analyzer tool (www.cibiv.at/software/pda) following the approach of Trovao et al. (2015). This down-sampling approach was repeated for each subtype stratified by host traits. (DOCX) [file ppat.1010062.s005.docx]
