# Supplementary material for: Ecological divergence of wild birds drives avian influenza spillover and global spread
Source: PLoS Pathog. 2022 May 19;18(5):e1010062. doi: 10.1371/journal.ppat.1010062 (PMC9119557; doi:10.1371/journal.ppat.1010062)
Supplement: Attachment — Submitted filename: Response to reviews_Plos_20220323.docx [file ppat.1010062.s008.docx]

Nichola J. Hill

Assistant Professor

Department of Biology

Integrated Science Complex

University of Massachusetts, Boston

___________________________________________________________

March 23, 2022

**Re: Response to reviewers**

Dear Editors,

I appreciate the time and effort that the reviewers and editors have taken to provide careful and constructive feedback on our manuscript. This is no small task in the middle of a pandemic, and I and my co-authors are genuinely grateful for these efforts.

We have adopted almost all the suggestions by the three reviewers and believe the paper is much improved as a result. We have addressed issues of sampling bias and dedicated additional paragraphs in the results and discussion to a fuller, more candid explanation of how these may influence the findings of the study. We have toned down some of the language around the quantitative, albeit descriptive findings that were not statistically tested. Lastly, we have added a discussion of the serological analyses that offer additional interpretations related to primary or prior exposure to influenza.

In summary, I believe we have addressed all the comments raised by the reviewers, and look forward to a final recommendation regarding whether the manuscript is suitable for publication. I hope this manuscript will make a relevant and timely contribution to the field in view of the recent movement of highly pathogenic avian influenza (HPAI) into North America. The current outbreaks in North America are causing outbreaks in poultry and wild birds of unprecedented scale and magnitude. I hope this translates to a renewed interest in studies that seek to clarify the natural history, epidemiology and evolution of influenza in wild and agricultural hosts.

Sincerely,

Nichola Hill, PhD

**Part 1 – Summary**

Reviewer #1:

“There are a handful of statements which I found slightly speculative and should be addressed either with further analysis or caveats to the text.”

> Yes, this is important point raised by the reviewer. We have attempted to address these concerns by changing the language in the results and discussion to avoid speculation (further details below: Part III - Minor issues). For example, the results heading (line 222) we have changed from: “Geographic distribution of HPAI relies on proximity to east Asia” to “*Geographic distribution of HPAI associated with proximity to endemic hotspots”.* We do feel that the data visuals are useful to the reader to make their own interpretation, and in many cases are more informative than quoting statistical correlations and p-values given the nature of the data (genomic surveillance not ecological). For the example above, we intentionally organized the X-axis of Figure 1 to reflect the spatial relationships among regions, so the reader can scrutinize how proximity to endemic hotspots (ie. East Asia) is associated with occurrence of HPAI H5.

Reviewer #2:

“My main concern regards the possible bias in the sequence data available, which, in my opinion, has not been sufficiently highlighted in the discussion. In particular, for the HPAI H5 it is important to consider that surveillance activity in wild birds is never or rarely performed in some countries (i.e. some African and Asian countries).”

> We agree. Our paper should go further to highlight that influenza surveillance is geographically uneven and that we lack coverage in Africa and Asia. We have added an additional paragraph to the Discussion (lines 466-479) to highlight these issues.

“*One of the limitations of this study was the dependence on publicly-available sequences that are inherently biased with regard to host or geographic region. For instance, active surveillance of wild birds is rarely performed in some African and Asian countries [**22**], resulting in less robust metrics for wild bird-mediated dispersal in these HPAI-endemic regions. We attempted to account for this in our downsampling strategy by including an equivalent number of samples for host taxa or geocluster when analyzing each subtype. However, the degree to which the subsequent analysis was sensitive to uneven sampling effort remains unclear. This may have led to over-estimates of the role of domestic geese in the wavefront expansion of the HPAI H5 virus relative to wild birds.”*

“Another possible bias the authors should consider is that in most cases surveillance in wild birds is only passive. The authors should take into account that HPAI H5 infections may have different outcomes in different wild bird species and there may be a sampling bias towards the more susceptible species…“Moreover, considering that several studies (including this one) indicate wild birds as the major cause of virus spread and introduction in new territories, as well as the major source of the virus for poultry, I was trying to figure out if it is correct to include viruses from poultry in the wavefront analysis.”

> Agreed, excellent point. We have revised the Discussion (line 474-479) to reflect this:

*“In addition, where wild bird surveillance is conducted there is a sampling bias towards sick or dead birds, or species that are more susceptible rather than resilient or asymptomatic birds. This highlights a critical need to invest in active surveillance of wild and domestic birds concurrently, rather than focus on poultry in HPAI-endemic regions, as these interfaces represents a critical interface for viral spillover, and an opportunity to learn about the changing role of wild birds in HPAI epidemiology.”*

Reviewer #3:

“The manuscript is mostly well written, however a major weakness of the study is that the limitations associated with sequence data (gaps in surveillance)…”

> Agreed. This point is also raised by Reviewer 2. Our paper should go further to highlight the gaps in influenza surveillance and we have added a paragraph to the Discussion (lines 466-479) as mentioned above:

*“One of the limitations of this study was the dependence on publicly-available sequences that are inherently biased with regard to host or geographic region. For instance, active surveillance of wild birds is rarely performed in some African and Asian countries [**22**], resulting in less robust metrics for wild bird-mediated dispersal in these HPAI-endemic regions. We attempted to account for this in our downsampling strategy by including an equivalent number of samples for host taxa or geocluster when analyzing each subtype. However, the degree to which the subsequent analysis was sensitive to uneven sampling effort remains unclear. This may have led to over-estimates of the role of domestic geese in the wavefront expansion of the HPAI H5 virus relative to wild birds.”*

“…as well as the antigenic analysis is not discussed adequately.”

> Agreed. This is a great point. A potential limitation of the antigenic analysis is that the exposure history of the birds is unknown. This is addressed below in Part II.

**Part II – Major Issues: Key Experiments Required for Acceptance**

Reviewer 3:

“The authors analyse serum from Alaskan gulls and ducks with unknown infection history to assess reactivity to a panel of H13 and H16 antigens from different clades. When a serum reacted with viruses from different subtypes the authors assume this as cross-reactivity between the strains. However, can the authors exclude that the serum contents of a bird were elicited through infection from only one subtype? Suggest to address the limitations to this analysis in the Discussion.

> Agreed. A potential limitation of the antigenic analysis is that the exposure history of the birds is unknown. These were field-collected samples, not from an experimental setting where exposure history could be controlled and manipulated. We attempted to account for this by only using very young birds in the analysis. This is stated in the Methods (line 630-633): “*Only sera from flighted juveniles (between 2-6 months) were assayed in order to maximize the likelihood that antibodies were de novo (not materially-derived) and were generated against first IAV exposure.”* However, we have also added a section to the Discussion (line 505-512) to clarify that the exposure history of the birds investigated in the study is unknown:

*“A limitation of the antigenic analysis is that the exposure history of the birds was unknown, therefore we cannot exclude that antibodies were elicited through infection from more than one subtype. We attempted to account for this by only including young birds (2-6 months) to maximize the likelihood that antibodies were de novo (not materially-derived) and generated against first IAV exposure. Future studies that aim to investigate cross-protective immunity in wild settings may benefit from longitudinal sampling of early life stages when birds have restricted mobility, or sampling of birds in human-modified environments where the possibility of recapture may be higher due to year-round supply of anthropogenic resources.”*

**Part III – Minor Issues: Editorial and Data Presentation Modifications**

Reviewer #1:

“One recurring issue is cases where patterns in the data are visually suggestive, but no formal analysis is presented to link the observed data to the broader biological explanation presented. Examples are results related to proximity to east Asia (~L200), the section on competitive exclusion (~L226), and the negative correlation (or regression?) between wavefront distance and diffusion rate for which statistics are alluded to, but not shown. Please tone down the language in these sections and in the discussion to be consistent with the speculative nature of the evidence, provide more robust analyses, or make verbal arguments for why no alternative explanations are possible.”

> We’ve taken care in the manuscript to avoid language that would suggest these specific biological relationships have been statistically tested. For example, we never use “significantly”, which is commonly associated with frequentist statistics that reference a p-value. We do feel however that the data visuals, although qualitative, are useful to the reader to make their own interpretation, and in many cases are more compelling and informative than quoting statistical correlations and p-values given the nature of the data (genomic surveillance not ecological). However, we agree that the reviewer raises an important point about ‘toning down the language’ and therefore we have made the following changes:

> For the results heading (line 222) we have changed: “Geographic distribution of HPAI relies on proximity to east Asia” to “*Geographic distribution of HPAI associated with proximity to endemic hotspots”.* For this point, we intentionally organized the X-axis of Figure 1 to reflect the spatial relationships among regions, so the reader can scrutinize how proximity to endemic hotspots (ie. East Asia) is associated with occurrence of HPAI H5.

> For the results heading (line 252), we have changed: “Competition and co-existence explains subtype distribution” to “*Competition and co-existence underlies subtype distribution*”. Switching the verb to “underlies” hopefully conveys that these antigenic mechanisms may contribute to but does not fully account for the distribution for subtypes observed, as was implied by “explains” which is more definitive.

> For the results section (line 359), we have changed: “For HPAI H5, a negative correlation between wavefront distance and diffusion rates was observed” to “*For HPAI H5, an inverse relationship was suggested between wavefront distance and diffusion rates*”.

“L43. Statement on extending models of viral evolution is unclear. It would be nice to explain in what way these models were extended. This isn’t clear in the later text either.”

> Yes, this was a challenge to convey concisely. The testing/extending of viral evolutionary models mentioned (line 42-44) in the Abstract is brief by necessity due to word count limit. Later in the Abstract (line 54) we say: “*Both neutral (geographic expansion) and non-neutral (antigenic selection) evolutionary processes were found to shape subtype evolution which manifested as unique geographic hotspots for each subtype at the global scale*”. We elaborate upon this in the Introduction (line 128): “*The circulation of multiple subtypes in wild birds that are either host-associated (H13, H16) or more host-generalist (HPAI H5) provides a unique opportunity to test and extend models of IAV evolution.”* We return to address this in the results (line 288-301) under the heading: “*Subtypes evolve by antigenic selection and geographic structuring*”. In the Discussion (line 497-500) we state: “*Prior models have proposed that the evolution of RNA viruses, including IAV, hinges on two dynamics: selective forces resulting from cross-immunity and non-selective epidemiological forces resulting from spatial structuring (Grenfell, 2004)...”* and again (line 519): “*Our results support that the mechanism of competition between and within subtypes is two-fold, driven by immune selection, as well as geographic isolation. This adds nuance to the view that antigenic selection acting on H13 and H16 is weak, relative to the pattern of geographic isolation and periodic inter-hemispheric transmission (Verhagen et al., 2020).*” We hope that this helps to highlight that our evidence for extending evolutionary models is woven throughout the manuscript, even though as the reviewer rightly points out, the first mention of this in the Abstract is brief.

“L103. If gulls are already known to have a key role in inter-hemispheric spread of IAVs, what is new about the new question being posed here? Is this statement specific to H13 and H16? Similarly in next sentence, explaining what exactly the ‘pressing need’ is (i.e., what would better understanding of IAV ecology enable which is not currently possible?) would convey significance more clearly.”

> The idea that gulls play a key role in the inter-hemispheric spread of IAVs stems from disparate studies with a smaller geographic and temporal scope (we cite them in the text). Whether this observation is reproducible at the global-scale and over a 10-year time frame is the focus of our paper. As an author, I usually try to refrain from writing styles that are combative or highlight shortcomings of other studies because the competitive culture needs to change. The field has advanced due to all these studies, both big and small. In agreement with the reviewer that the significance of the global scale could be made clearer, we have made the following change (line 105): “A consistent finding of surveillance studies *conducted at local or regional-scales* has been that gulls play a key role in the inter-hemispheric spread of IAV [16, 17] attributed to their long-distance pelagic movements [18, 19]. There is a pressing need to compare the roles of avian reservoirs, *using longer term, globally distributed data* to account for variation in IAV ecology.

L145. A brief summary of the dataset/study design at the start of the results or towards the end of the introduction would be helpful to understand the results section. (I gave up half way through the results and skipped to the methods). For example, number of viruses sequenced per subtype and per bird species; whether the timescale and geographic provenance of samples was comparable across subtypes; and how the samples were collected (active/passive sampling?).

> Agreed, this is a good idea. I’m going to blame the trend in formatting Methods after Results/Discussion. I don’t love it either! I’ve added a section to the first line of the results (line 160) to help provide context without making the manuscript too repetitive: “*Wild birds were live-caught and sampled across North America between 2008-2018 and the 888 influenza sequences generated (including H13: n=23 sequences and H16: n=58 sequences) were compared with global reference sequences for the three subtypes.”* The methods are described in more detail later in the manuscript (line 574). The specific capture and sampling details can be found in Hill et al., 2016 (waterfowl) and Ineson et al., 2021 (gulls) that are included in the References.

“L153. The difference between H13 and H16 in terms of multiple clade circulation is difficult to see in Fig S5. It appears that representatives of all 3 clades of H16 were found in the most recent year, indicating sustained transmission. For H13, clade 2 is only found in Continental Europe and clade 1 seems to have gone extinct, which is inconsistent with statements in the text related to continued detection of all 3 clades. Please clarify how these conclusions were reached or revise the text.”

> Good catch, the H13 and H16 trees in the supplementary (Fig S5) got mixed up when importing to the Word document. Apologies/Thank you! That is genuinely embarrassing. Error fixed. Also, we did try to make the trees of sufficiently high image quality for Fig S5 so that readers can zoom in and see these relationships. I expanded the image size to help.

“L162. ‘same avian reservoir’ is misleading. Surely the different subtypes infect bird species at different rates. It seems plausible that these infections of birds with different host life histories influence viral evolutionary rate. Similar issue on L174 – you are dealing with a community of hosts, not multiple subtypes adapted to the same host species, or is cross-species transmission so rare that it can be ignored? I suggest a clarification here.”

> Agreed, this has been revised to help clarify that “same avian reservoir” is specifically in reference to H13 and H16 in gulls. We have changed this sentence (line 178) to: “*These results indicate that the evolutionary dynamics of IAV subtypes can be unique and distinct even when hosted by the same avian reservoir, such as H13 and H16 in gulls.”* Our subsequent analysis (Figure 3 - A and B) provides strong support for gulls acting as the definitive host reservoir for these two subtypes.

“L221: Transmission between North America and Eurasia was consistently rare for all 3 subtypes, relative to transmission within each region”

> Agreed. This sentence (line 241) has been changed to: “*Transmission between North America and Eurasia was consistently rare for all 3 subtypes, relative to transmission within each region*”

“L245. It would be interesting to overlay the dynamics of viral effective population size for the two H16 clades separately in Fig 2C to see if they mirror the expected epidemiological dynamics, but I understand this is considerable work and not vital to the conclusions of the study, so fine with whatever the authors decide.”

> I love this idea! I think that would make an excellent addition to the next paper related to the transmission dynamics in Alaska. Comparing the viral evolutionary dynamics side-by-side with local-scale epidemiology deserves more attention, I fully agree. For the current paper, this is beyond the scope of what we were aiming to do. But this is a great suggestion for future analyses.

“L268. Perhaps a discussion point, but isn’t it possible that the lack of introductions from South America to Alaska reflects bird migratory patterns more than cross-reactivity? This seems more consistent with the data given that South American H13 viruses have no problem dispersing to other areas of North America (Fig. 1) where presumably there are other H13 and H16 viruses with which it might cross-react. A broader statistical analysis of pairwise cross reactivity vs spatiotemporal overlap (e.g., proportion of years where each pair of strains overlapped in each location where both was reported at least once) might be a better way to formally test if cross reactivity is affecting viral invasion success, but even that would just be a correlation.”

> This is a good point, but perhaps not straight-forward to test with our study design. Alaska is quite isolated and unlikely to be representative of the lower 48 states in the US where viruses from South America can periodically occur. Also, banding data from the Blue-winged Teal suggests that they frequently over-winter in Central America, providing a mechanism for influenza to spread between the two continents. Given the lack of bird movement data from this region, I’m not sure it’s clear-cut with regard to bird migration. I think both mechanisms together (antigenic cross-reactivity and bird migration) are plausible to explain viral dynamics, but we didn’t explicitly analyze bird movement data.

“L282. By “host barriers” do you mean physiological or ecological (ie, ducks and gulls don’t commonly live/forage in the same habitats)? Please clarify.”

> The use of “host barriers” here intentionally doesn’t specify the mechanism, which is beyond what these antigenic analyses can realistically test. There are many biotic and abiotic factors that contribute to host barriers that may limit transmission of virus between bird taxa in nature. In our view, it would stray into speculation to list the possible reasons why these host barriers exist. The important part is that the antigenic analyses support that host barriers exist in the first place, and this is complementary to the viral evolutionary analysis.

“L290. Provide the denominator for the percentages of host jumps.”

> The denominator ie. the total number of jumps exceeds 100,000 and is an unwieldy number to report. The number is so large because these counts are individual jumps summed over the length of each branch (>10 years) and then added together over the entirety of the tree. The convention with Markov jumps is to just report % for this reason; the raw data is at a scale that isn’t especially useful for the reader. I would be happy to provide the Markov jump data for each subtype (H5, H13, H16) as supporting material if the reviewer/editor feel this is helpful.

“L516. Provide sample sizes for birds caught, samples tested etc.”

> The focus of this paper is the viral sequence data (all of which is available in Genbank), not the field collection effort which has been previously reported, along with prevalence analyses in Hill et al., 2016 and Ineson et al., 2021. This paper doesn’t address prevalence (positives/total number sampled birds) or incidence in the wild bird reservoir. Therefore, to limit overlap with published work, we have chosen not to include a lengthy description of the field collection effort for this specific manuscript. We believe it isn’t necessary for interpretation of the findings in the current paper and may even serve as a distraction.

“L555. Presumably WGS was done on the amplified virus not field collected samples, but be explicit about this.”

> Yes, correct. We have changed this sentence (line: 590) to: “*Whole-genome sequencing was performed on the DNA amplicons at the J. Craig Venter Institute in Rockville, MD”*. In addition, we have added additional text and references to clarify the Illumina sequencing methods and platform used.

“L600. Numbers don’t add up to 888 sequences unless you had 807 H5 sequences. Please clarify.”

> The 888 sequences collected over 10 years represent a wide range of HA subtypes, beyond H13 and H16. The majority were H3 and H4 from Alaskan ducks that we analyzed in Hill et al., 2016. Agreed that this sentence is a little confusing. Therefore, we have changed this sentence to reflect the total number of subtypes detected (line 663): “*A total of 888 complete influenza sequences representing 12 HA subtypes were generated from our North American surveillance between 2008-2018, including H13 (n=23 sequences) and H16 (n=58 sequences).”*

“L625. Sentence on 2005-2018 sequences not being downsampled is unclear. Are you saying that tree tips, not their underlying sequences were downsampled or that only sequences from certain years were excluded from downsampling?”

> Agreed. This sentence needs to be clarified. We were trying to say that sequences were stratified and downsampled by region, but not by year. Some viral strains/subtype appear or disappear according to year due to competition or drift. Those are legitimate evolutionary processes that we wanted to capture in the dataset. We have changed this sentence (line 692): *“Samples were limited to 2005-2018 but were not stratified and downsampled by year as the spatio-temporal distribution was considered an important feature linked to the epidemiology of each subtype.”* In contrast, uneven sampling effort according to geographic region is far more common (as discussed by reviewer 2) so we felt compelled to account for this by stratifying and downsampling.

“L679. Does the host-specific geographic expansion somehow account for the possibility that multiple species/host groups could have contributed to spread? If, not independent spread from the MRCA to the current range limit by each host group seems unrealistic. The lines below suggests that latitude and longitude data are incorporated and I see how that might work for a host specific diffusion rate, but please elaborate if/how it corrects for the issue specifically for the wavefront distance metric.”

> Yes, correct. The host-specific estimates include multiple species or hosts within that taxonomic group (ie. gulls vs geese), not just individual birds. So this is a collective estimate across an entire taxa, consequently the wavefront distances can be really large. We have changed this sentence to clarify (line 758): “*The host-specific diffusion rate (km/year) is the average speed the virus travels within a host group, and represents a collective estimate made up of multiple individuals and species*”.

“Fig 1. Need to define the lines and shading in the right column (maps)”

> Agree. The caption for Fig 1 has been changed to: “*The global distribution of each subtype is depicted by shading on the maps (right panel). Lines indicate the route of inter-hemispheric virus flow and represent the branching pattern of internal and external nodes of the underlying phylogenetic tree.”*

Reviewer #2:

“Line 153. Figures should be numbered according to the order of appearance in the text.”

> Agreed. The order of the figures in the supporting material has been corrected.

“Line 160. Replace “result” with “results””

> Agreed.

“Lines 163-165. Please, provide a brief explanation in the introduction of the classification system used for the HPAI H5 of the Gs/GD lineage.”

> Agreed. We have changed the sentence (line 119) to: “*The global expansion of the HPAI H5 subtype, originating in domestic geese in 1996 in Guandong, China (GsGD lineage) [**20**] represents a phenotype of IAV with distinctly different evolutionary dynamics from H13 and H16. Viral descendants of A/goose/Guangdong/1/1996 belonging to the HPAI H5 subtype have achieved persistent circulation in domestic poultry and wild ducks with periodic spillover to mammals [**21-23**]*. We’ve added 3 additional citations that describe in detail the original GsGD 1996 outbreak.

> In addition, we have changed the sentence (line 181): “*The evolution of HPAI H5 indicates extensive diversification over time consistent with the known clade designations determined by the WHO/OIE [**28**] resulting in the most recent clade 2.3.4.4*”. This includes a reference to the WHO/OIE working group so that readers can refer to the most recent HPAI H5 classification system.

“Line 164. Clade 2.3.4.4 expanded not only into continental Europe, but also in Africa and several Asian countries.”

> Agreed. This sentence (line 183) now reads: “*the most recent clade 2.3.4.4 (95% HPD: February 2009 – April 2011) that expanded into continental Europe, Asia and Africa and a rare introduction event into North America…”*

“Line 168. The range “November 2014-July 2015” is not correct (2004-2005?). Please modify.”

> Corrected. Line 187 has been updated.

“Lines 635-637. It is not clear if the datasets were analysed separately and for which analysis each dataset was used. I suggest naming the dataset and mentioning for each analysis the dataset used.”

> Yes, each of these datasets was analyzed separately. This sentence (line 702) has been changed to: “*Each of the subtype data sets were analyzed separately. Analyses were performed to assess spatial spread according to geoclusters: H13: n=398, H16: n=191, HPAI H5: n=295. Separate analyses were performed to assess spread according to host taxa: H13: n=298, H16: n=202, HPAI H5: n=367.*

Reviewer #3:

“1. The antigenic panel in Figure 2 is named with location of origin. For consistency, consider include the clade numbers shown in Fig S5.”

> We respectfully disagree. The primary message for the antigenic analysis relates to geographic origin of the viruses and the figure has been formatted and color-coded accordingly. Adding the clade numbers from the supporting figure adds a second dimension that may have little value to the reader. Unfortunately Figure 2 is already a busy figure.

2. The language surrounding MRCA/origins can be cleaned up a little throughout the ms. For example, “H16 consisted of two geographically-distinct clades (Northern Europe and Mississippi-Atlantic US).” should be “originated/emerged from two …”

> Agreed. The language has been changed in line with the reviewer’s recommendations.

“3. Lines 238-240. “but rapidly accrued with the introduction of the South American clade, as inferred from Bayesian skyride plots that showed changes in viral effective population size over time.” is not apparent from Figure 2B. Instead, there appears to be a gradual increase in relative genetic diversity until 2015, followed by fluctuations.”

> Apologies. The effective population size may appear more gradual that what it really is due to the smoothing means applied in the Tracer software. The H13 subtype was not detected in 3 years prior (2012-2014), or the one year after the peak in viral diversity observed in 2015. This is evident in Figure 2 and confirms that the gradual build is an artefact of the smoothing factor applied. We hope that the correlation between the introduction of the South American lineage and the sudden increase in effective population size (No) is still apparent to the reader. Probably the best we can do to illustrate this despite the constraints of the software.

“4. Suggest to rephrase Line 81 “as relative extremes on the (avian) phylogeny” is not clear.”

> Agreed. This sentence (line 85) has been changed to: “*These two avian orders are highly diverged on the avian phylogeny, with Anseriformes estimated to be an older, ancestral lineage (97.6 million years) while Charadriiformes are a more recently evolved lineage (79.5 million years) based on multi-locus phylogenetic dating*”

“5. In Fig 2 legend clarify the dashed lines used in Fig 2 F and G.”

> To address this I removed the dashed lines. I’m not sure they were adding much anyway!

6. It would be useful it the clade numbers used in Fig S5 were incorporated in the description of the viruses, rather than just MRCA.
> We respectfully disagree. The primary message for the antigenic analysis relates to geographic origin of the viruses. Adding the clade numbers from the supporting figure adds a second dimension that may have little value to the reader.

8. Avoid “very in Discussion Line 369, or describe the magnitude of differences.

> Agreed. This sentence (line 402) has been changed and “very” removed.

9. Minor typo “out (of) necessity”

> Agreed. This sentence has been changed and “of” added.
